# Arachnoid Cyst as a Late Complication of Selective Dorsal Rhizotomy: A Case Report

**DOI:** 10.1055/a-2482-9156

**Published:** 2024-12-23

**Authors:** Maya T. van Noort, Paul van Schie, K. Mariam Slot, Laura A. van de Pol, Annemieke I. Buizer, Vincent de Groot

**Affiliations:** 1 Department of Rehabilitation Medicine, Amsterdam University Medical Center, Amsterdam, The Netherlands; 2 Department of Neurosurgery, Amsterdam University Medical Center, Amsterdam, The Netherlands; 3 Department of Child Neurology, Amsterdam University Medical Center, Amsterdam, The Netherlands; 4 Emma Children's Hospital, Amsterdam University Medical Center, Amsterdam, The Netherlands; 5 Amsterdam Movement Sciences, Rehabilitation & Development, Amsterdam University Medical Center, Amsterdam, The Netherlands

**Keywords:** arachnoid cyst, case report, cerebral palsy, complication, selective dorsal rhizotomy

## Abstract

**Background and importance** Selective dorsal rhizotomy (SDR) is a surgical technique to treat spasticity, mainly in children with spastic cerebral palsy (CP). In this report, a unique case of a late arachnoid cyst, causing radiating pain in the left leg, is presented. This is relevant to clinicians managing the long-term follow-up of patients who underwent selective dorsal rhizotomy (SDR).

**Clinical presentation** A 25-year-old male with bilateral spastic CP, who underwent SDR at the age of 7, presented with symptoms of progressive radiating pain in the left leg. Magnetic resonance imaging (MRI) revealed the presence of a large arachnoid cyst and a remarkable dorsal position of the cauda equina. After dissection of the cyst, the previously experienced radiating pain immediately subsided; however, the patient developed urinary retention and constipation. Cauda compression was ruled out by MRI. The constipation subsided quickly, and the patient performed self-catheterization until 1 month postoperatively for the urinary retention after which there were no signs of ongoing bladder dysfunction.

**Conclusion** Arachnoid cyst formation can be a late complication of SDR and can cause lumbosacral radicular syndrome in the late postoperative course in select cases.

## Background and Importance

Selective dorsal rhizotomy (SDR) is a surgical intervention that reduces spasticity in children with spastic cerebral palsy (CP). The primary goal is to improve walking and gross motor skills. The surgical procedure involves identifying the dorsal roots of L2–S1 with the use of intraoperative neuromonitoring and interrupting the reflex arc by selective ablation of 25 to 50% of these roots. 1 2 In a systematic review, Mishra et al 3 identified common long-term structural complications of SDR such as scoliosis and hyperlordosis. Other long-term spinal abnormalities include kyphosis and spondylolysis. 4 Although arachnoid cysts are a known complication in surgeries that involve opening the dura (as are pseudomeningocele and scar tissue formation), their occurrence as a complication after SDR has not been reported. 5 In this report, a patient with CP is presented who underwent SDR at the age of 7 and developed a large symptomatic arachnoid cyst as a late complication at the age of 25. This case study is reported in line with the SCARE Criteria. 6

## Clinical Presentation

**Fig. 1:**
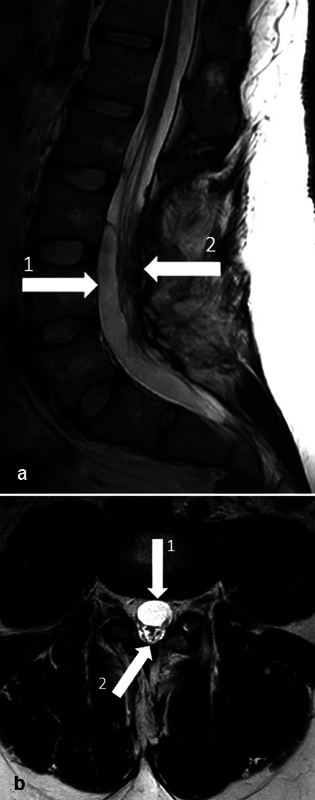
Preoperative magnetic resonance imaging (MRI) of lumbosacral spine. ( **a** ) Sagittal view. 1, arachnoid cyst; 2, cauda equina. ( **b** ) Axial view. 1, arachnoid cyst; 2, cauda equina.

The patient is a 25-year-old male, with a history of CP. He was born after an uncomplicated pregnancy through a Caesarean section, because of breech position. The neonatal period was normal and psychomotor development in the first year was described as slightly delayed. At the age of 10 months, the patient developed an increased tension in the muscles of the left leg, with diminished mobility, causing difficulty with standing. On magnetic resonance imaging (MRI) of the brain at the age of 2 years, periventricular leukomalacia and a porencephalic cyst in the right hemisphere were found. The patient was diagnosed with asymmetric bilateral spastic CP, Gross Motor Function Classification System (GMFCS) II. After several treatments with botulinum injections to improve his gait, the patient was counseled for SDR at the age of 7. During the procedure, laminoplasty from L2 to L5 was performed, after which the dura was opened and the L2 to S1 roots were identified on both sides. Motor and sensory branches were separated. After neuromonitoring, 30 to 60% of the sensory branches from L2 to S1 were ablated bilaterally, with the preservation of the bladder function related fascicles in S1. After hemostasis and watertight closure of the dura, the laminae were reinstated en bloc and parent surfaces were closed. The postoperative course was normal, and the clinical condition improved with intensive physical therapy and ankle foot orthoses. There was no evidence of postoperative infection or trauma. Eventually the child was able to walk without aids and his walking distance was not limited. He was discharged from follow-up at the age of 15. After 10 years, the patient presented at our outpatient clinic with significant back pain radiating to the calf and sole of the left foot, since 3 months. The pain persisted in all positions, intensifying during walking and under increased pressure. Subsequent spinal MRI findings showed an arachnoid cyst extending from L3 to S2, and a remarkable dorsal position of the cauda equina ( Fig. 1 ). After shared decision-making and obtaining informed consent, a surgical fenestration of the arachnoid cyst was performed.

### Surgical Technique and Clinical Course

The patient was positioned in prone position. The laminae in the old laminoplasty were removed, which was challenging because of extensive formation of scar tissue in the epidural plane. After adequate exposure of the dura, an ultrasound was performed, which showed the upper and lower pole of the arachnoid cyst. Then, a midline durotomy was performed. The nerves of the cauda equina were abnormally tightly adherent to the dura and each other, probably as a result of extensive postoperative arachnoiditis. After lengthy dissection and intradural adhesiolysis, the nerves were mobilized to the left side, although they remained attached to the posterior wall of the arachnoid cyst. The lateral wall of the arachnoid cyst was then excised throughout the entire length. New ultrasound did not show any remaining cyst. The dura was then closed in a watertight fashion. In the postoperative phase, the previously experienced radiating pain immediately resolved. However, the patient developed urinary retention and constipation. While exploring the patient history it became clear that the patient had idiopathic urinary hesitancy since young age. Besides one episode of pain above the symphyses while urinating at the age of 8 years, there were no signs of pre-existing impaired bladder function. At examination both the perianal sensation and rectal tone were intact, making cauda equina involvement less likely. To rule out potential postoperative complications such as epidural hemorrhage and involvement of the cauda equina, an MRI of the lumbar spine was performed. The imaging showed a significant reduction in the size of the arachnoid cyst, and no compression of the cauda equina. The constipation subsided quickly, and the patient performed self-catheterization until 1 month postoperatively for the urinary retention. Apart from the pre-existing urinary hesitancy, there were no signs of ongoing bladder dysfunction. The patient started to experience back pain radiating to the left foot 2 months after the surgery. MRI was consistent with postoperative imaging, showing a remaining heterogenous collection in the laminectomy plane. Since the postoperative period of clinical improvement was so short, new fenestration of this secondary arachnoid cyst was refrained from. Implementation of a cysto-arachnoid drain has been performed, after which the radiating pain subsided. 5

## Discussion

SDR is an effective treatment for reduction of spasticity of the lower extremity muscles in young children. 7 This report presents a unique case of a late and severe complication of this procedure. SDR followed by arachnoid cyst formation is not documented in literature. Moreover, symptomatic secondary intradural spinal arachnoid cysts (SAC) occurring after neurosurgical procedures are also rare, with only 33 iatrogenic cases identified in a systematic review between 1990 and 2022 by Wang et al. 8 Notably, none of the reported cases were secondary to SDR but often due to epidural anesthesia. The current case highlights the need for clinicians to be aware of potential late arachnoid cyst formation after SDR, especially when these patients present with radiating pain or other signs of nerve compression. We suggest performing imaging of the lumbosacral spine in an early stage after the onset of symptoms in these patients. In addition, clinicians should inform patients who underwent SDR about potential temporary bladder dysfunction when considering new lumbosacral spinal surgery, even if bladder function was preserved during the initial SDR. Moreover, considering the high rate of recurrence (51%) in iatrogenic SAC, 8 patients need adequate postoperative monitoring and follow-up after cyst fenestration.

## Conclusion

Arachnoid cyst formation can be a late complication of SDR and can cause lumbosacral radicular syndrome in the late postoperative course in select cases.
